# Combinatorial Analysis of Circulating Biomarkers and Maternal Characteristics for Preeclampsia Prediction in the First and Third Trimesters in Asia

**DOI:** 10.3390/diagnostics12071533

**Published:** 2022-06-23

**Authors:** Willie Lin, Sen-Wen Teng, Tzu-Yi Lin, Ronald Lovel, Hsin-Yu Sung, Wen-Ying Chang, Tang Bo-Chung Wu, Hsuan-Yu Chen, Le-Ming Wang, Steven W. Shaw

**Affiliations:** 1 Meridigen Biotech Co., Ltd., Taipei 114, Taiwan; willie.lin@meridigen.com (W.L.); linus.tang@meridigen.com (T.B.-C.W.); 2 Department of Obstetrics and Gynecology, Cardinal Tien Hospital, New Taipei 231, Taiwan; senwen1955@yahoo.com.tw; 3 School of Medicine, Fu-Jen Catholic University, New Taipei 242, Taiwan; 4 College of Medicine, Chang Gung University, Taoyuan 333, Taiwan; forever841230@gmail.com; 5 Meribank Biotech Co., Ltd., Taipei 114, Taiwan; lovel.ronald@gmail.com (R.L.); dora.sung@meribank.com.tw (H.-Y.S.); nancy.chang@meribank.com.tw (W.-Y.C.); sherry.chen@meribank.com.tw (H.-Y.C.); 6 Department of Obstetrics and Gynecology, Wan Fang Hospital, Taipei 116, Taiwan; lemingmd90@gmail.com; 7 Graduate Institute of Clinical Medicine, Taipei Medical University, Taipei 110, Taiwan; 8 Department of Obstetrics and Gynecology, Taipei Chang Gung Memorial Hospital, No. 199, Dun-Hua North Road, Taipei 105, Taiwan

**Keywords:** miR-181a, miR-210, miR-223, risk algorithm, sFlt-1/PlGF

## Abstract

We aim to establish a prediction model for pregnancy outcomes through a combinatorial analysis of circulating biomarkers and maternal characteristics to effectively identify pregnant women with higher risks of preeclampsia in the first and third trimesters within the Asian population. A total of two hundred and twelve pregnant women were screened for preeclampsia through a multicenter study conducted in four recruiting centers in Taiwan from 2017 to 2020. In addition, serum levels of sFlt-1/PlGF ratio, miR-181a, miR-210 and miR-223 were measured and transformed into multiples of the median. We thus further developed statistically validated algorithmic models by designing combinations of different maternal characteristics and biomarker levels. Through the performance of the training cohort (0.848 AUC, 0.73–0.96 95% CI, 80% sensitivity, 85% specificity, *p* < 0.001) and the validation cohort (0.852 AUC, 0.74–0.98 95% CI, 75% sensitivity, 87% specificity, *p* < 0.001) from one hundred and fifty-two women with a combination of miR-210, miR-181a and BMI, we established a preeclampsia prediction model for the first trimester. We successfully identified pregnant women with higher risks of preeclampsia in the first and third trimesters in the Asian population using the established prediction models that utilized combinatorial analysis of circulating biomarkers and maternal characteristics.

## 1. Introduction

Preeclampsia (PE) is a gestational disease that begins after the 20th gestational week. This disease was characterized by the presence of high blood pressure and proteinuria accumulating over 300 mg in 24 h, thrombocytopenia < 100,000/µL, elevated liver transaminases (>twice of normal values), pulmonary edema, new-onset visual or cerebral disturbances [1]. In addition, PE may cause fetal growth restriction, premature rupture of the placenta, and even fetal death without active treatment. The current method in resolving PE is to deliver the fetus with the placenta. Therefore, precise delivery timing was critical to guarantee the safety of the mother and neonate [2].

PE screening in the first trimester, as recommended by physicians, allows adequate prevention to decrease risk [3]. Previous studies showed that aspirin treatment before the 16th gestational week can significantly reduce PE incidence by 60% and delay the development of PE [4,5]. Furthermore, specific biomarkers have been reported to improve PE screening [6]. Nowadays, fetal abnormalities are examined using non-invasive prenatal testing (NIPT) by detecting circulating nucleic acid molecules [7].

Vascular dysfunction and trophoblast immaturity serve as the main causes of preeclampsia [8,9]. sFlt-1 could bind vascular endothelial growth factor (VEGF) and placental growth factor (PlGF) to reduce blood vessel growth. MicroRNA (miRNA), a non-coding small RNA molecule, represents an alternative factor in detecting PE, as it affects the proliferation and differentiation of the trophoblast [10]. To begin with, strong expression of miR-210, a hypoxia-inducible factor [11,12], was noted in maternal serum and placental tissue in PE [13]. Another miRNA, miR-181a, was reported to be significantly higher in the placenta and plasma in PE [14,15]. In addition, the upregulation of miR-181a is associated with the downregulation of AKT-serine and threonine, which are critical players in the insulin signaling pathway [16]. The miRNA profiling assay demonstrated that the level of miR-223 was low in PE [17]. Similarly, a high-throughput assay showed a low concentration of miR-223 in early-onset PE [18]. Moreover, the ratio of an anti-angiogenic factor, soluble fms-related receptor tyrosine kinase 1 (sFlt-1), to an angiogenic factor, placental growth factor (PlGF), would increase under PE causing improper development of blood vessels [19,20]. An sFlt-1/PlGF cut-off ratio of thirty-eight demonstrated benefit in predicting late-onset PE in Asian women, reducing unnecessary hospitalizations and economic burden for women with suspected PE [21,22]. To improve pregnancy outcomes with timely treatment, a new predictive model should be developed for early diagnosis. Although some miRNA and proteic markers showed a good PE detection rate, the combination of these markers or the addition of other biomarkers may improve predictivity [23,24]. Therefore, this study aimed to examine the expression levels of miR-181a, miR-210, miR- 223 and the sFlt-1/PlGF ratio in the serum of pregnant women in the first and third trimesters to evaluate their individual and combinatorial screening efficacies.

## 2. Materials and Methods

### 2.1. Study Design

**Figure 1:**
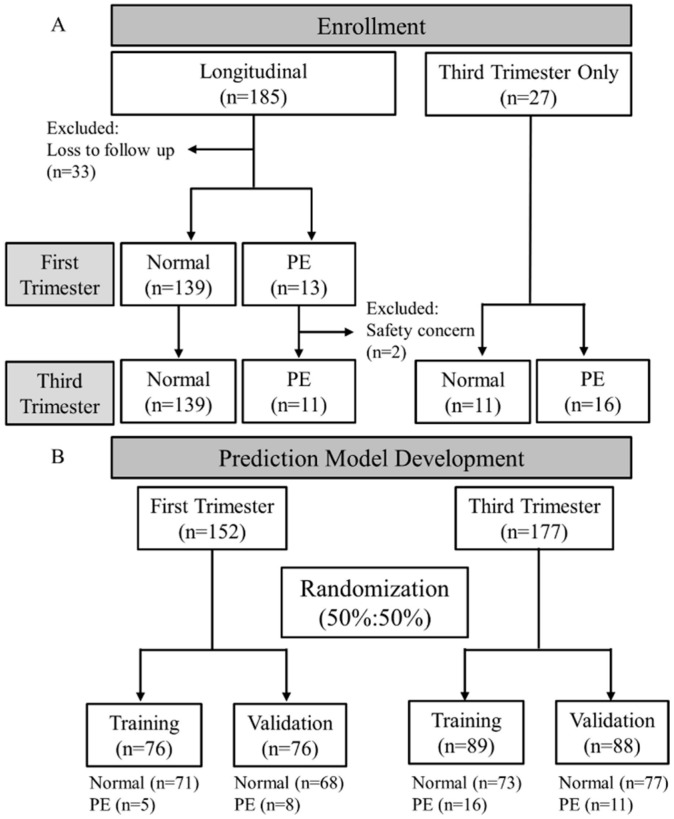
Flowchart of the (**A**) Enrollment methods for sample collection and (**B**) Prediction model development.

A total of 212 pregnant women were recruited between 2017 and 2020 (Figure 1A). Initially, 27 pregnant women whose pregnancy outcomes had been determined by physicians in the third trimester were enrolled, and none of them were suspected or confirmed for preeclampsia. Later on, 185 pregnant women were enrolled at the check-up in the first trimester and followed-up subsequently until delivery. The inclusion criteria included 20 to 45-year-old women with singleton or twin pregnancies between the 10th and 40th gestational week. The pregnancy status of the longitudinal study participants was followed up and recorded from the first trimester until the third trimester. The physician determined the pregnancy outcome as normal or preeclampsia for the 3rd trimester-only participants. All participants were willing to give consent. Exclusion criteria excluded participants with HBV, HIV I/II, Syphilis or other diagnosed infectious diseases; exhibited typical symptoms of Kaposi’s sarcoma on the skin or disseminated lymphadenopathy; experienced postpartum, miscarriage or abortion within six months; had been quarantined; with a mental disorder, cancer or diabetes diagnosis; or under conditions deemed unsuitable for blood sampling by the doctor. Written consent was obtained from all pregnant women participating in this study. Based on the definition from ACOG, preeclampsia was diagnosed by preexisting or pregnancy-associated hypertension (>140/90 mmHg) with any one of proteinuria, thrombocytopenia, impaired liver function, severe persistent right upper quadrant or epigastric pain, renal insufficiency, pulmonary edema, headache and visual disturbances [25].

The study was approved by the Institutional Review Boards of Chang Gung Medical Foundation (201701517B0), Tri-Service General Hospital (1-105-05-148), Cardinal Tien Hospital (CTH-106-2-4-066) and the Joint Institutional Review Board of Dianthus MFM Clinic (17-003-A-1). All procedures were performed in line with the principle of the Declaration of Helsinki. All subjects gave their informed consent for inclusion before they participated in the study.

### 2.2. Establishment of the Prediction Model

Blood samples included in the first-trimester prediction model were acquired from 152 pregnant women followed up longitudinally. Two cases with PE were delivered before the samples were obtained in the third trimester and thus were excluded from the following processes. Blood samples included in the third-trimester prediction model were acquired from 27 women determined with or without PE and 150 women with ongoing pregnancy (Figure 1). The amount of protein and miRNAs from each subject sample were analyzed and the maternal characteristics were recorded. Various biomarkers combination was calculated, and the combination with the highest AUC was chosen for algorithm development. The prediction model for both the first and the third trimesters was developed by randomly dividing the sample size by 50%/50% to create a Training cohort (71 normal with 5 PE and 73 normal with 16 PE) and a Validation cohort (68 normal with 8 PE and 77 normal with 11 PE) (Figure 1B).

### 2.3. Blood Sample Collection

Whole blood was drawn into a 9-mL Serum Clot Activator Tube (CAT, Greiner Bio-One, Kremsmunster, Austria) and centrifuged at 500× *g*, 20 min, 4 °C to obtain serum, aliquoted, and stored at −80 °C until analyzed.

### 2.4. miRNA Isolation and Reverse Transcription

Total RNAs were extracted from serum using miRNeasy Serum/Plasma Advanced Kit (Qiagen, Hilden, Germany) according to the manufacturer’s instructions. For sample normalization, Caenorhabditis elegans miR-39 (cel-miR-39) was added to each sample [26]. Ten μL of total RNA was reverse-transcribed using Transcriptor First Strand cDNA kit (Roche, Mannheim, Germany) and miRNA-specific stem-loop primer (Genedragon, Taipei, Taiwan). cDNA was stored at −20 °C until analyzed.

### 2.5. Mature miRNA qPCR Analysis

cDNA expression was amplified with QuantStudio 5 qPCR machine (Thermo Fisher, MA, USA) using LightCycler 480 SYBR Green I Master (Roche, Mannheim, Germany) and specific forward miRNA primers and universal reverse miRNA primers (Genedragon, Taipei, Taiwan). All samples were assayed in triplicate. Each miRNA expression was normalized to cel- miR-39 and calculated as 2^−^^ΔCt^, ΔCt = (Ct[miR]−Ct[cel-miR-39]) [26].

### 2.6. Enzyme-Linked Immunosorbent Assay

Serum concentration levels of PlGF (R&D Systems, Minneapolis, MN, USA, DPG00) and sFlt-1 (R&D Systems, Minneapolis, MN, USA, DVR100C) were measured using a sandwich ELISA kit. Standards and samples were run in duplicate and were performed according to the manufacturer’s instructions.

### 2.7. Statistical Analysis

Both the relative expression levels of the miRNAs and the sFlt-1/PlGF ratio were normalized to log10. Comparison of miRNAs relative expression level Multiple of Median (MoM) and sFlt-1/PlGF ratio MoMs between normal and PE group were analyzed with Mann–Whitney U-test. Characteristics of the study population were analyzed using Student’s *t*-test or Fischer exact test for continuous or categorical variables, respectively. Pearson correlation was used to study the correlation between biomarkers and BMI. By utilizing the IBM SPSS program, samples from the first or the third trimesters were randomly divided into two equal numbered sample sizes to create Training and Validation cohorts. Binomial logistic regression analysis of the Training cohort was used to generate a predictive model for PE in the first or the third trimester and was further challenged in the Validation cohort. The area under curve (AUC), along with the sensitivity and specificity were calculated using GraphPad Prism to determine the performance of the prediction model on both the Training and Validation cohorts. The optimal cut-off point was determined by the highest Youden Index. *p* < 0.05 was considered statistically significant.

### 2.8. Data Sharing Statement

The first and third-trimester raw data, as well as the training and validation data set reported in this article, have been deposited in a public repository database, BioStudies under the following accession number: S-BSST753.

## 3. Results

### 3.1. Characteristics of the First-Trimester Study Population

One hundred and thirty-nine women experienced a normal pregnancy, and 13 developed PE in all cases. Maternal characteristics and pregnancy outcomes were documented (Table 1). Notably, first trimester BMI, systolic and diastolic blood pressure, and proteinuria are significantly higher, while the gestational age and newborn weight were significantly lower in the PE group. No significant difference was observed in the age factor.

### 3.2. First-Trimester Biomarker Analysis and Screening Method Comparison

**Figure 2:**
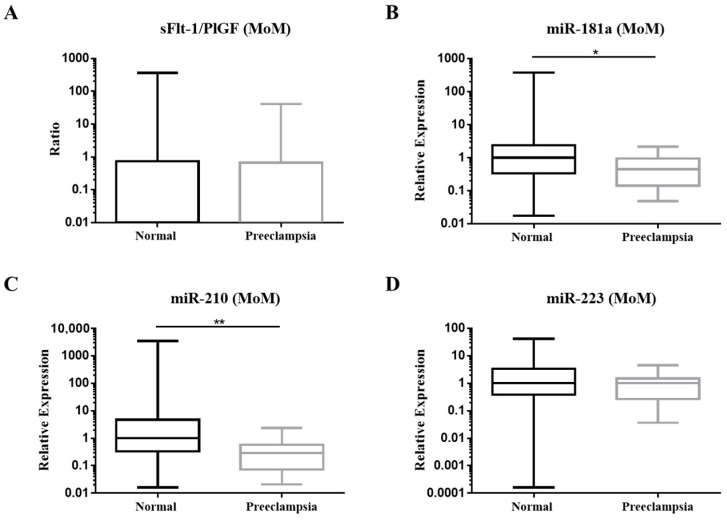
Box plots of the multiple of median (MoM) values of first trimester (**A**) sFlt-1/PlGF; (**B**) miR-181a; (**C**) miR-210 and (**D**) miR-223. Comparisons between normal (*n* = 139) and PE (*n* = 13) group were calculated by Mann–Whitney U test, * *p* < 0.05, ** *p* < 0.01.

**Figure 3:**
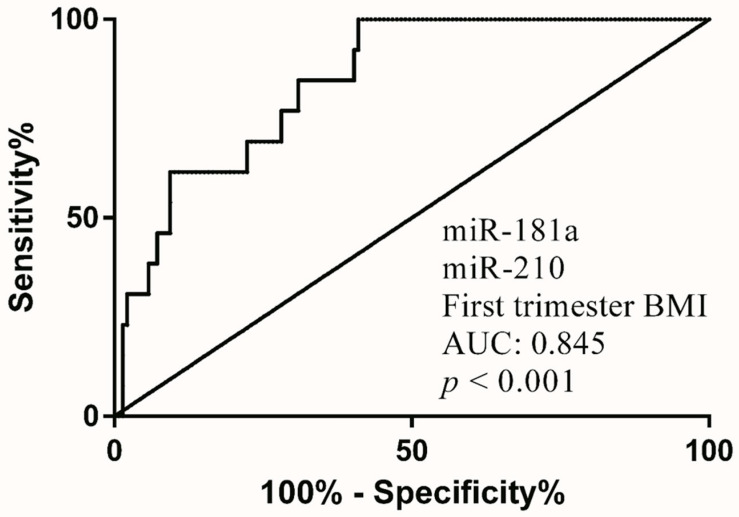
Receiver operating characteristics (ROC) curve of PE combinatorial screening model for First trimester (combination of miR-181a + miR-210 + first trimester BMI) to screen whether the pregnancy is at risk of developing preeclampsia or not. AUC, area under curve.

MoM of miR-181a and miR-210 were significantly lower in the PE group, yet no significance was observed in MoM of sFlt-1/PlGF ratio and miR-223 (Figure 2). Therefore, various combinations of biomarkers were analyzed for the validity of PE prediction. The sensitivity, specificity and area under curve (AUC) are presented in Table 2. In particular, BMI achieved the best performance in prediction (Table 2). Together, first-trimester PE prediction screening with miR-210, miR-181a and BMI demonstrated the greatest AUC (0.845) (Figure 3) along with a sensitivity of 62% at 10% FPR (False positive rate) (Table 2).

### 3.3. Characteristics of the Third-Trimester Study Population

Maternal characteristics and pregnancy outcomes were documented (Table 3). Consistent with the results from the first-trimester study population, the PE group showed significantly higher third-trimester BMI, systolic and diastolic blood pressure and proteinuria. In contrast, the gestational week and newborn weight were significantly lower. However, no significant difference in the age factor was observed.

### 3.4. Third-Trimester Biomarker Analysis and Screening Method Comparison

**Figure 4:**
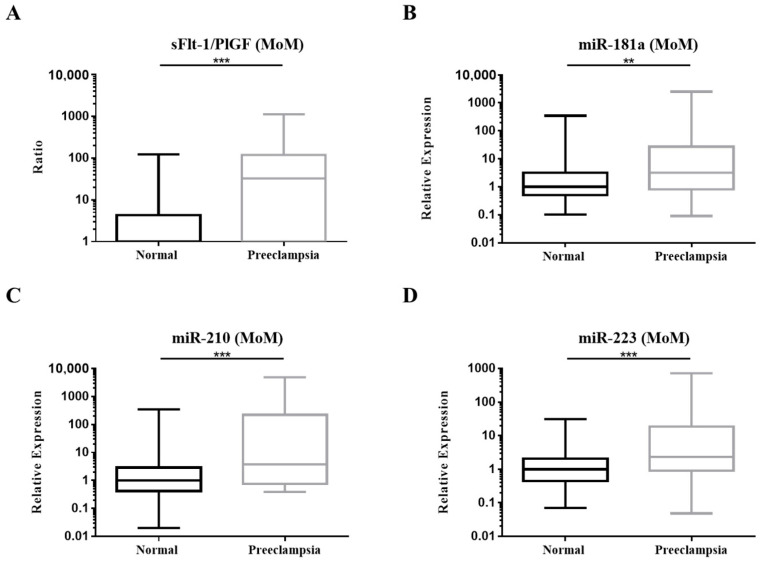
Box plots of the multiple of median (MoM) values of third-trimester (**A**) sFlt-1/PlGF ratio; (**B**) miR-181a; (**C**) miR-210 and (**D**) miR-223. Comparisons between the normal (*n* = 150) and the PE (*n* = 27) group were calculated by Mann–Whitney U test, ** *p* < 0.01, *** *p* < 0.001.

**Figure 5:**
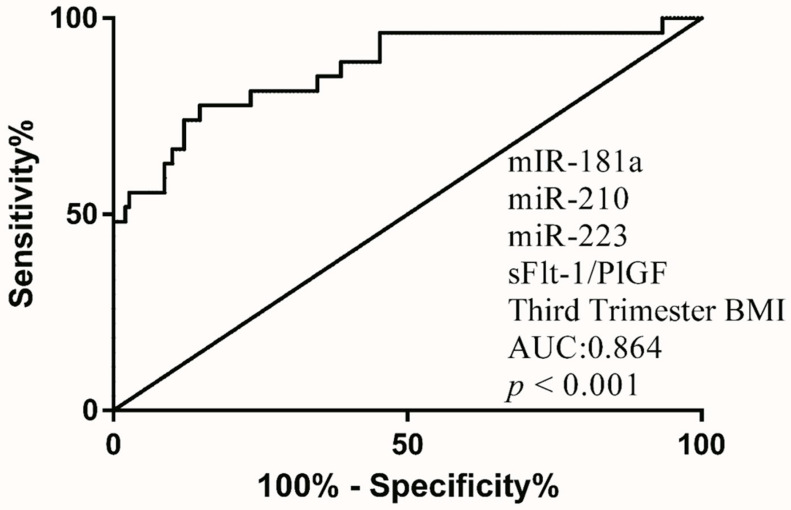
Receiver operating characteristics (ROC) curve of PE combinatorial screening model for Third trimester (combination of miR-181a + miR-210 + miR-223 + sFlt-1/PlGF ratio+ third- trimester BMI) to screen whether said pregnancy is at risk on developing preeclampsia or not. AUC, area under curve.

Higher MoM of the sFlt-1/PlGF ratio, miR-181a, miR-210 and miR-223 were observed in the PE group (Figure 4). Various combination of biomarkers was analyzed for the validity of PE prediction. Combination of sFlt-1/PlGF MoM, miR-181a MoM, miR-210 MoM, miR-223 MoM and BMI achieved the highest AUC (0.864) (Figure 5 and Table 4).

### 3.5. The Development of PE Prediction Models for the First and the Third Trimesters

The first-trimester PE prediction model was developed by training cohort with miR-181a, miR-210 and first trimester BMI. The performance at cut-off value of >0.09 was 0.848 AUC, 0.73–0.96 95% CI, 80% sensitivity, 85% specificity, 27% PPV, 98% NPV and *p* < 0.01. (Figure S3) Validation cohort with 68 normal and 8 PE cases demonstrated 0.852 AUC, 0.74–0.98 95% CI, 75% sensitivity, 87% specificity, 40% PPV, 97% and *p* < 0.01 at >0.09 cut-off value (Table 5). The third- trimester PE prediction model was developed by training cohort with the sFlt-1/PlGF ratio, miR-181a, miR-210, miR-223 and third-trimester BMI. The performance at cut-off value >0.18 showed 0.852 AUC, 0.73–0.98 95% CI, 69% sensitivity, 93% specificity, 68% PPV, 93% NPV and *p* < 0.001 (Figure S3). The validation cohort demonstrated 0.886 AUC 0.886, 0.77–1.00 95% CI, 64% sensitivity, 92% specificity, 53% PPV, 95% NPV and *p* < 0.001 (Table 5), indicating optimistic performance of both prediction models.

Cut-off value of PE prediction models for both trimesters was picked from the highest Youden index. The prediction model to calculate the risk of PE during the first and the third trimesters were shown as the following.

First-trimester PE prediction:

Y = −5.398 + (0.051) × miR-181a MoM + (−0.349 × miR-210 MoM) + 0.120 × first-trimester BMI(1)

Third-trimester PE prediction:

Y = −4.233 + 0.021 × sFlt-1/PlGF ratio MoM + −0.085) × miR-181a MoM + 0.045 × miR-210 MoM + 0.139 × miR-223 MoM + 0.062 × third-trimester BMI(2)

PE probability = 1/(1 + Euler’s Constant^−Y^)

## 4. Discussion

### Principal Findings

Our study demonstrated that specific factors express significantly in the first and third trimesters in the Asian population. A significant difference in BMI between the PE and the normal group was observed in the first and third trimesters. Consequently, BMI served as an essential factor in the prediction model. Correlation among obesity, miR-181a, and miR-210 was reported in previous studies. Adipose tissue would express lower miR-181a but higher miR-210 in the placenta of pregnant women with high BMI [27,28,29]. In this study, we revealed a strong positive correlation between miR-181a and miR-210 in both the first and third trimesters (Figure S1), suggesting that these factors might be involved in the pathophysiology of PE.

## 5. Results

In vitro evidence using HTR8/SVneo—a trophoblast cell line—showed inhibition of miR-181a and miR-210 could increase cell invasion ability [30,31]. Therefore, we hypothesized that the decrease of both miRNAs in the first trimester resulted in aberrant trophoblast invasion and pregnancy complications, which should be further verified. Interestingly, we observed that the sFlt-1/PlGF ratio was only effective for the third-trimester prediction. The combination of the sFlt-1/PlGF ratio, miRNA and BMI showed a higher AUC value than the ratio alone. Therefore, if a higher sensitivity is preferred, a combination variety of sFlt-1/PlGF and miRNAs should be considered (Table 4). Based on a previous study performed by Chaemsaithong et al., a certain model predicting the risk of preeclampsia was compared in the Asian population [32]. The screening from the ACOG showed a detection rate of 54.6% and a false-positive rate of 20.4, and the screening from the NICE showed a detection rate of 26.3% and a false-positive rate of 5.5%. However, these two models merely utilized maternal characteristics and medical history to detect the high-risk group of preeclampsia. The screening from the Fetal Medicine Foundation achieved detection rates of 48.2%, 64.0%, 71.8% and 75.8%, at 5%, 10%, 15% and 20% fixed false-positive rates. Nevertheless, mean arterial pressure, uterine artery doppler and serum concentration of placental growth factor should be measured to capture further data. Our screening model simplifies the procedure of screening for preeclampsia.

## 6. Clinical Implications

Studies have emphasized the need to include racial origin as a consideration. 

For every prediction algorithm, a correction was needed for each ethnicity different from the majority used during the algorithm development [33,34]. Chaemsaithong et al. showed that the Asian population had lower mean arterial pressure and PlGF compared to the Caucasian population due to anthropometric differences. An Asian-specific PE prediction is required to provide a more reliable outcome.

## 7. Research Implications

Since the study was conducted in Taiwan, and the samples primarily originated from the Asian population, the capability of the prediction model for application in Asia in this study is reinforced. Notably, an increase in the incidence of PE in Taiwan was observed from 2001 to 2014, showing a rising need for PE screening [35]. Studies for third-trimester PE prediction had concluded that utilizing the sFlt-1/PlGF ratio in clinical practice could decrease the economic burden of pregnant women due to its ability to rule out preeclampsia, therefore decreasing unnecessary hospitalization [35]. We also observe the same pattern in this study, as our third-trimester PE prediction model had a much higher NPV than its PPV (Table 5).

## 8. Strengths and Limitations

Our study demonstrated a good performance of these biomarkers for PE screening. Nevertheless, in precedence, applying further challenges to an independent cohort with statistically adequately powered sample size is the key to validating and increasing the efficacies of both PE prediction models for future clinical use. However, the information regarding the use of low-dose aspirin, which may decrease the risk of preeclampsia, was not retrieved in this study. The actual event rate could be overestimated.

## 9. Conclusions

Here we presented the first study that utilized the reported biomarkers, such as miR-181a and miR-210, in a combinatorial analysis to identify populations afflicted with high PE risks. This is also a landmark step in providing precise screening to allow early intervention for improving maternal and fetal outcomes and a groundbreaking effort in developing PE prediction models for pregnancies in the first and third trimesters.

## Figures and Tables

**Table 1:** First-trimester maternal characteristics and pregnancy outcomes of the study population.

	Normal (*n* = 139)	PE (*n* = 13)	*p*
**Maternal Characteristics**			
Age, year, mean (SD)	32 (3.2)	34 (4.0)	0.15
BMI, kg/m^2^, mean (SD)	24.1 (5.7)	29.8 (4.5)	<0.001
**Pregnancy outcomes**			
Systolic Blood Pressure, mmHg, mean (SD)	127 (12.2)	153 (15.7)	<0.001
Diastolic Blood Pressure, mmHg, mean (SD)	75 (12.2)	90 (12.2)	<0.001
Gestational Week, week, mean (SD)	38 (2.0)	36 (1.2)	<0.001
Newborn Weight, g, mean (SD)	3050 (678.1)	2718 (365.6)	<0.05
Proteinuria, *n* (%)	20 (14%)	8 (62%)	<0.001

Data are given as mean. The comparisons between the control and the PE group were calculated using the Student’s *t*-test for continuous variables or Fischer exact test for categorical variables. SD: Standard deviation.

**Table 2:** First-Trimester Preeclampsia Screening Comparison.

Screening Method	AUC	95% CI	10% FPR	Cut-Off
sFlt-1/PlGF Ratio MoM	0.504	0.34–0.67	0%	NA
miR-181a MoM	0.686	0.56–0.82	23%	0.1393
miR-210 MoM	0.761	0.65–0.88	31%	0.1377
miR-223 MoM	0.584	0.45–0.72	8%	0.0658
BMI	0.809	0.70–0.92	39%	NA
miR-210 MoM + BMI	0.838	0.74–0.94	54%	NA
miR-210 MoM + miR-181a MoM + BMI	0.845	0.76–0.93	62%	NA

10% FPR: 10% False positive rate, 95% CI: 95% Confidence interval, AUC: Area under curve, BMI: Body mass index, MoM: Multiple of median, NA: Not applicable.

**Table 3:** Third-trimester maternal and pregnancy characteristics of the study population.

	Normal (*n* = 150)	PE (*n* = 27)	*p*
**Maternal characteristics**			
Age, year, mean (SD)	32 (4.0)	34 (4.9)	0.09
Third trimester BMI, kg/m^2^, mean (SD)	25.8 (4.5)	28.7 (5.7)	<0.01
**Pregnancy outcomes**			
Systolic Blood Pressure, mmHg, mean (SD)	130 (16.2)	157 (21.8)	<0.001
Diastolic Blood Pressure, mmHg, mean (SD)	76 (11.8)	94 (15.3)	<0.001
Gestational Week, week, mean (SD)	38 (1.2)	36 (2.9)	<0.001
Newborn Weight, g, mean (SD)	3030 (375.3)	2632 (722.2)	<0.001
Proteinuria, *n* (%)	25 (17%)	17 (63%)	<0.001

Data are given as mean. Comparisons between normal and PE group were calculated by Student’s t test for continuous variables or Fischer exact test for categorical variables. SD: Standard deviation.

**Table 4:** Third-Trimester Preeclampsia Screening Comparison.

Screening Method	AUC	95% CI	10% FPR	Cut-Off
sFlt-1/PlGF Ratio MoM	0.739	0.61–0.87	63%	NA
miR-181a MoM	0.667	0.54–0.79	37%	7.690
miR-210 MoM	0.719	0.61–0.83	44%	7.136
miR-223 MoM	0.709	0.59–0.83	41%	5.128
BMI	0.645	0.52–0.77	33%	NA
Protein + BMI	0.795	0.69–0.90	59%	NA
Protein + miR-181a MoM	0.791	0.68–0.90	56%	NA
Protein + miR-210 MoM	0.806	0.70–0.91	59%	NA
Protein + miR-223 MoM	0.831	0.73–0.93	67%	NA
Protein + miRNAs	0.856	0.76–0.95	70%	NA
Protein + miRNAs + BMI	0.864	0.78–0.95	67%	NA

10% FPR: 10% False positive rate, 95% CI: 95% Confidence interval, AUC: Area under curve, miRNAs: miR-181a MoM + miR-210 MoM + miR-223 MoM, MoM: Multiple of median, Protein: sFlt-1/PlGF Ratio MoM, NA: Not applicable.

**Table 5:** First- and third-trimester preeclampsia training & validation cohort analysis.

Cohort	Cut-Off Value	AUC	95% CI	Sensitivity	Specificity	PPV	NPV	*p*
First trimester								
Training	>0.09	0.848	0.73–0.96	80%	85%	27%	98%	<0.01
Validation		0.852	0.74–0.98	75%	87%	40%	97%	<0.01
Third trimester								
Training	>0.18	0.852	0.73–0.98	69%	93%	68%	93%	<0.001
Validation		0.886	0.77–1.00	64%	92%	53%	95%	<0.001

95% CI: 95% Confidence interval; AUC: Area under curve, NPV: Negative predictive value, PPV: Positive predictive value.

## Data Availability

The data that support the findings of this study are available from the corresponding author upon reasonable request.

## Supplementary Materials

The following supporting information can be downloaded at: https://www.mdpi.com/article/10.3390/diagnostics12071533/s1, Figure S1: Correlation between miR-181a and miR-210 expression during (A) First trimester (r: 0.9826, *p* < 0.001) and (B) Third tri-mester (r: 0.9519, *p* < 0.001). Correlation was determined by Pearson’s correlation coefficient; Figure S2: Receiver operating characteristics (ROC) curve with respectively AUC values of each screening model for First trimester. AUC: area under curve; Figure S3: Receiver operating characteristics (ROC) curve with respectively AUC values of each screening model for Third trimester. AUC: area under curve.
