# Supplementary material for: Combinatorial Analysis of Circulating Biomarkers and Maternal Characteristics for Preeclampsia Prediction in the First and Third Trimesters in Asia
Source: Diagnostics (Basel). 2022 Jun 23;12(7):1533. doi: 10.3390/diagnostics12071533 (PMC9320107; doi:10.3390/diagnostics12071533)
Supplement: Supplementary file 1 [file diagnostics-12-01533-s001.zip › diagnostics-1713314-supplementary.pdf]

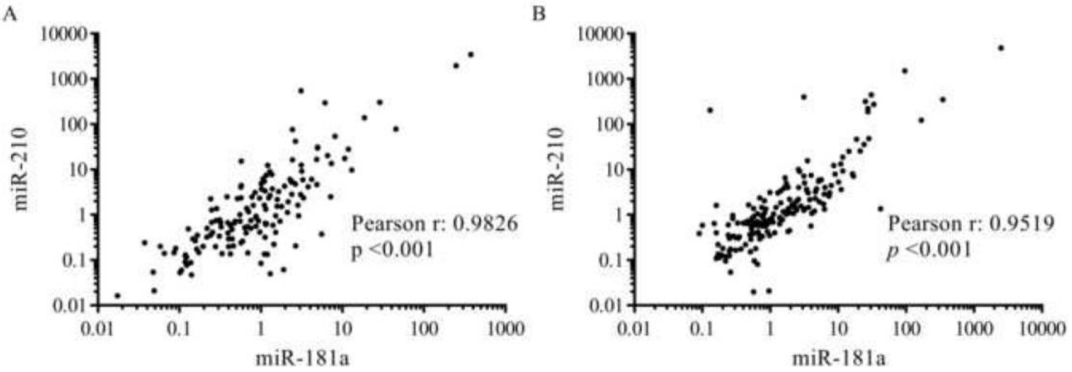

Figure S1 Correlation between miR-181a and miR-210 expression during (A) First trimester (r: 0.9826, $p < 0.001$) and (B) Third trimester (r: 0.9519, $p < 0.001$). Correlation was determined by Pearson's correlation coefficient.

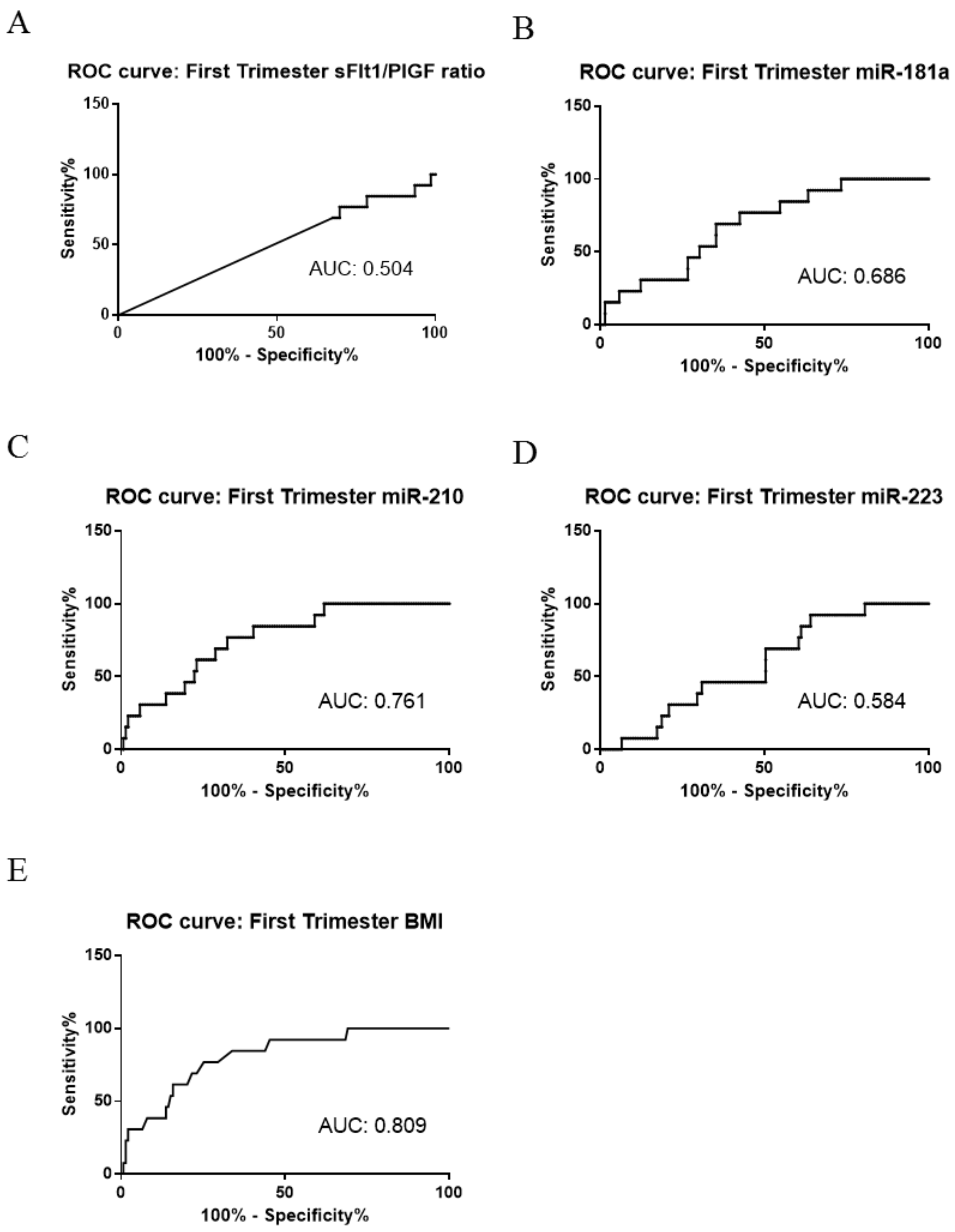

Figure S2 Receiver operating characteristics (ROC) curve with respectively AUC values of each screening model for First trimester (**A**) sFlt-1/PlGF; (**B**) miR-181a; (**C**) miR-210; (**D**) miR-223 and (**E**) BMI. AUC: area under curve.

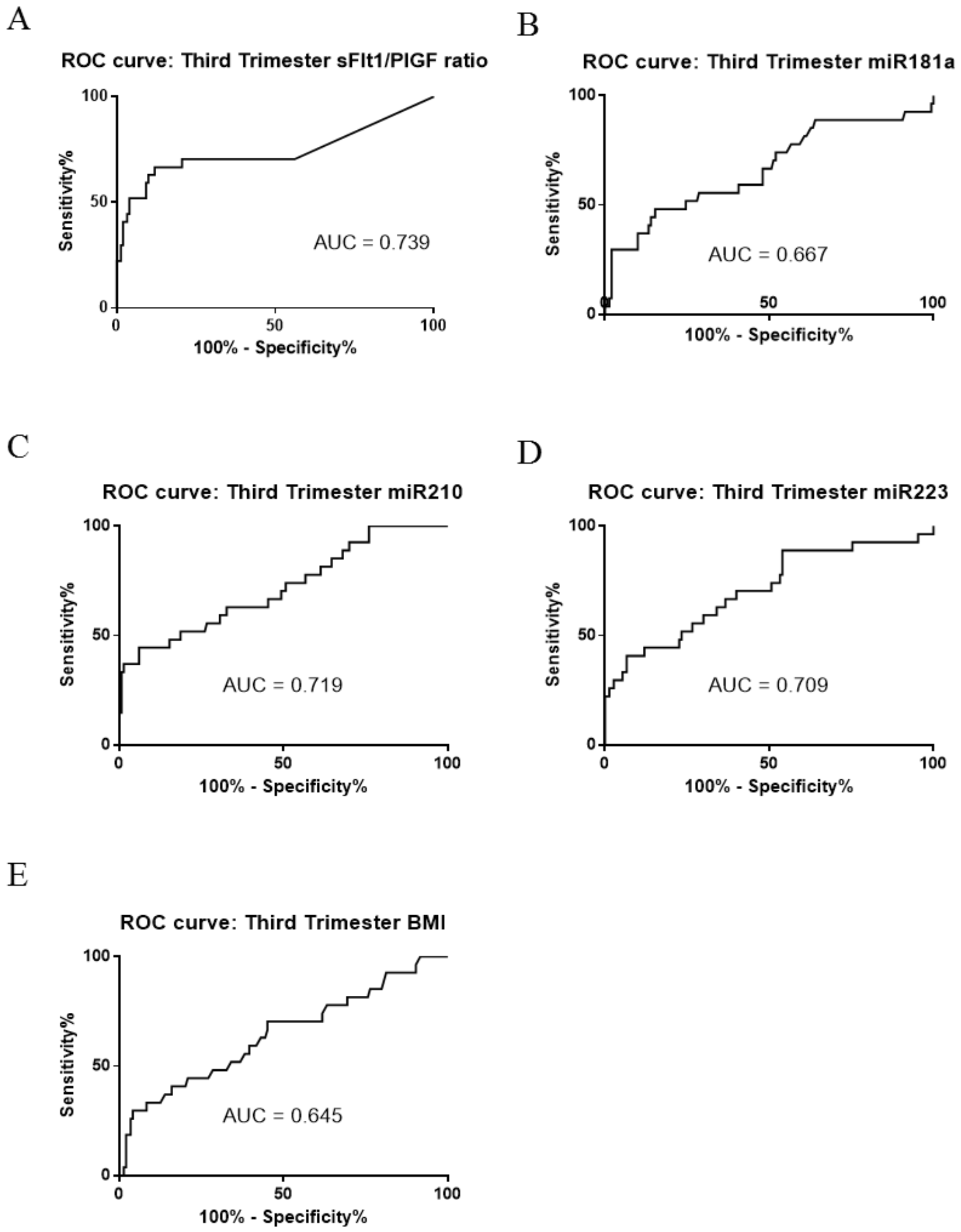

Figure S3 Receiver operating characteristics (ROC) curve with respectively AUC values of each screening model for Third trimester (**A**) sFlt-1/PlGF; (**B**) miR-181a; (**C**) miR-210; (**D**) miR-223 and (**E**) BMI. AUC: area under curve.
